# Impaired touch sensation on hairy skin in HCN3-deficient mice

**DOI:** 10.3389/fnins.2025.1697582

**Published:** 2026-01-12

**Authors:** Katharina Metzner, Tamara Hussein-Zahovic, Yomna Behery, Stefanie Fenske, Martin Biel, Achim Schmidtko

**Affiliations:** 1 Institute of Pharmacology and Clinical Pharmacy, Goethe University Frankfurt, Frankfurt am Main, Germany; 2 Department of Pharmacy, Center for Drug Research, Ludwig-Maximilians-Universität München, Munich, Germany

**Keywords:** dorsal root ganglia, HCN channel, mice, pain, sensory neurons, touch

## Abstract

The hyperpolarization-activated cyclic nucleotide-gated (HCN) channel HCN3 is expressed in sensory dorsal root ganglia (DRG) neurons, but its contribution to somatosensory processing remains poorly understood. Here, using RNA *in situ* hybridization, we found that *Hcn3* is widely expressed in various populations of DRG neurons. Analysis of HCN3-deficient mice in a series of behavioral tests for somatosensory function revealed that HCN3 deletion led to profound impairments in mechanical sensation on hairy skin. However, the mechanical sensation on glabrous skin and responses to noxious heat and cold stimuli were not affected in the absence of HCN3. Electrophysiological recordings revealed that deletion of HCN3 reduced the HCN current (I_h_) density and affected the action potential kinetics in thoracic (Th9–Th10) DRG neurons, which innervate hairy skin. However, electrophysiological parameters were unaltered in lumbar (L4–L5) DRG neurons. These findings suggest that HCN3 channels are specific regulators of low-threshold mechanoreceptors that innervate hairy skin.

## 1 Introduction

Hyperpolarization-activated cyclic nucleotide-gated channels, encoded by four genes (HCN1–4), are pore-forming membrane proteins expressed in a range of electrically excitable cells. They are activated by membrane hyperpolarization and conduct a depolarizing inward current carried by Na^+^ and K^+^ that contributes to the resting membrane potential and input resistance (Biel et al., 2009; He et al., 2014). Activation of HCN2 and HCN4 can be potentiated by cAMP via direct interaction with a cyclic nucleotide binding domain, whereas HCN1 and HCN3 have been reported to be relatively insensitive to cAMP binding (Sartiani et al., 2017). HCN channels are highly expressed in cardiac cells and in neuronal populations of the peripheral and central nervous systems (Sartiani et al., 2017). HCN4 is the main protein isoform that underlies the “funny” inward pacemaker current, I_f_, in the pacemaker region of the heart (Baruscotti et al., 2011; Sartiani et al., 2017). In neurons, the main isoforms driving inward hyperpolarization-activated current, I_h_, are HCN1, HCN2, and HCN3 (Momin et al., 2008).

HCN channels are also expressed in distinct populations of sensory neurons, which are the primary afferent neurons in the somatosensory system that convert thermal, physical, and chemical stimuli into electrical signals and convey these signals to the spinal cord. Previous studies have found that HCN1 is predominantly expressed in large- and medium-diameter neurons and in a population of cold-sensitive small neurons (Jiang et al., 2008; Moosmang et al., 2001; Orio et al., 2009). Global HCN1 knockout mice exhibited enhanced hairy skin sensitivity and deficits in texture discrimination (Orefice et al., 2019), reduced cold sensitivity on a cold plate (Orio et al., 2009), and attenuated cold allodynia after peripheral nerve injury (Momin et al., 2008). HCN2 has been reported to be the most abundant isoform in small nociceptive neurons and Aδ nociceptors (Emery et al., 2011; Momin et al., 2008; Tsantoulas et al., 2016; Tu et al., 2004). Tissue-specific knockouts lacking HCN2 in Na_V_1.8-expressing nociceptors showed normal acute pain thresholds but reduced inflammatory pain and considerably attenuated mechanical, heat, or cold pain following peripheral nerve injury (Emery et al., 2011; Schnorr et al., 2014), attenuated mechanical allodynia in a model of diabetic neuropathy (Tsantoulas et al., 2017), and ameliorated mechanical hypersensitivity in models of migraine (Tsantoulas et al., 2022). Based on these characteristics, HCN2 is considered a potential pharmacological target for the development of novel analgesics that are effective against neuropathic pain and migraine (Bernard Healey et al., 2021; Tsantoulas et al., 2016; Tsantoulas et al., 2022).

Unlike HCN1 and HCN2, the functional role of HCN3 in sensory neurons remains poorly understood. Single-cell RNA sequencing of sensory neurons has detected *Hcn3* mRNA in distinct cell populations of nociceptors (both non-peptidergic and peptidergic C fiber neurons) and in C- and Aδ-low-threshold mechanoreceptors (LTMR) (Zeisel et al., 2018; Zheng et al., 2019). A previous study using global HCN3-deficient (*Hcn3^−/−^*) mice reported that HCN3, despite its expression in nociceptors, plays only a minor role in pain processing. In particular, *Hcn3^−/−^* mice showed normal acute thresholds to heat or mechanical stimuli, unaltered inflammatory pain, normal mechanical allodynia, and thermal hyperalgesia after peripheral nerve injury, but reduced responses to a pinprick after peripheral nerve injury (Lainez et al., 2019). However, the function of HCN3 expressed in C-LTMR and Aδ-LTMR remains elusive. Both C-LTMR and Aδ-LTMR exclusively innervate hairy skin, where they form lanceolate endings associated with awl/auchene and zigzag hair follicles and contribute to touch sensations (Handler and Ginty, 2021; Li et al., 2011). Here, we further explored the possible functions of HCN3 in sensory neurons using tissue staining, behavioral analysis, and patch-clamp recordings. We report an unrecognized specific contribution of HCN3 to the sensation of touch on hairy skin.

## 2 Materials and methods

### 2.1 Animals

Experiments were performed on *Hcn3^−/−^* (Fenske et al., 2011) and wild-type (WT) mice of both sexes. Animals were housed on a 12 h light/dark cycle with access to food and water *ad libitum*. All experiments were reviewed and approved by the local Ethics Committee for Animal Research (Regierungspräsidium Darmstadt, Germany; approval number V 54–19 c 20/15–FR/1013). They adhered to the ARRIVE (Animal Research: Reporting on *In Vivo* Experiments) guidelines and conformed to the Directive 2010/63/EU guidelines. All efforts were made to minimize animal suffering and reduce the number of animals used.

### 2.2 Behavioral testing

All behavioral studies were performed during the light phase by observers blinded to the treatment of the animals. Experiments were performed on animals of both sexes. However, we did not analyze the effect of sex, as we were not powered to detect sex differences.

#### 2.2.1 Touch assays

For the tape response assay on hairy skin, mice were individually placed in Plexiglas cylinders and habituated for 5 min. A 3-cm piece of common laboratory adhesive marking tape (Diversified Biotech, United States) was placed on the dorsal fur of the mouse. A response was scored when the mouse bit the piece of tape or showed a visible “wet-dog shake” motion in an attempt to remove the tape. Responses occurring within 5 min of tape application were counted. A single test was performed for each animal (Gross et al., 2020).

For the cotton swab assay on hairy skin, mice were individually placed in Plexiglas boxes and habituated for 2 h. We used a cotton swab whose cotton tip was “puffed out” so that it was more than three times larger than normal and performed a < 1-s stroke along the dorsal fur (from rostral to caudal) five times, with a 10-s interval between. The number of unpleasant reactions after stroking the fur was recorded. An unpleasant reaction was defined as an escape response such as ducking or dodging. The frequency of unpleasant reactions was calculated as a percentage per mouse.

For the tape removal test on glabrous skin, the mice were individually placed in Plexiglas cylinders and habituated for 5 min. A 1-cm piece of a common laboratory adhesive marking tape (Diversified Biotech) was placed on the plantar side of the hindpaw. Latency time was measured until the mouse bit or the piece of tape was removed. The cutoff time was 2 min. Three tests per animal were used for analysis (Gautam et al., 2024).

For the cotton swab assay on glabrous skin, we used a cotton swab whose cotton tip was “puffed out” so that it was more than three times larger than normal (Garrison et al., 2012). The mice were placed on an enclosed elevated mesh and habituated for 60 min. We performed a < 1-s stroke along the plantar paw surface (from the heel to the toes) five times, with a 10-s interval between, and recorded the number of paw withdrawals. The frequency of withdrawal was calculated as a percentage per mouse.

For the von Frey filament test on glabrous skin, mice were placed in boxes on an elevated metal mesh floor and habituated for at least 30 min. Calibrated von Frey filaments ranging from 0.04 to 1.4 g (Ugo Basile, Italy) were applied to the hindpaw until they bowed for 1 s. Only obvious withdrawal responses (lifting, licking, or flinching of the paw) to the applied stimulus were recorded. Each filament was applied five times within 1 min, followed by a 1-min break, and then five additional applications within 1 min. Both hindpaws were measured equally. Response frequency was calculated as the percentage of withdrawal per mouse (Murthy et al., 2018).

#### 2.2.2 Pain assays

For the hot plate test, mice were individually confined in a Plexiglas chamber on a heated metal surface (Hot/Cold Plate; Ugo Basile, Italy). The time between placement and nocifensive behavior (shaking or licking of the hindpaw, jumping) was recorded, and the animal was removed from the plate immediately after a response. To prevent tissue damage, temperatures of 50, 52, and 54 °C were applied with cutoff times of 40, 30, and 20 s, respectively. A single test per animal per temperature was performed (Petersen et al., 2019).

For the tail immersion test, mice were immobilized in aluminum foil, which allowed free tail movement. For accommodation, the tip of the tail (approximately one-third of its length) was immersed in a water bath (Sunlab D-8810; NeoLab, Germany) at 32 °C for 20 s. Then, the tip of the tail was immersed in another water bath maintained at 46, 48, 50, or 52 °C with cutoff times of 80, 40, 20, or 10 s, respectively. The latency time to the tail withdrawal reflex was recorded, and the tail was removed from the bath immediately after the response. A single test per animal per temperature was used for the analysis (Mogil and Wilson, 1997).

For the cold plantar assay, mice were acclimated on a borosilicate glass plate (6.5 mm thickness; GVB GmbH, Germany) in transparent plastic enclosures and acclimated for 40–60 min. Powdered dry ice was packed into a 3-mL modified syringe (B. Braun, Germany) with a cut-off top (1 cm diameter). The open end of the syringe was held against a flat surface while pressure was applied to the plunger to compress the dry ice, and then the dense dry ice pellet was applied to the glass surface underneath the hindpaw. The latency to move the paw vertically or horizontally away from the glass plate was measured using a stopwatch. An interval of at least 7 min was allowed between testing separate paws of a single mouse, and an interval of at least 15 min was allowed between trials on any single paw. Three to five measurements were performed per paw (Luiz et al., 2019).

### 2.3 *In situ* hybridization

The mice were euthanized by CO_2_ inhalation and perfused with 4% formaldehyde (PFA) in phosphate-buffered saline (PBS) for 5 min. Lumbar (L4–L5) and thoracic (Th9–Th10) DRGs were dissected, post-fixed in 4% PFA for 10 min, incubated in 20% sucrose in PBS overnight, and embedded in tissue freezing medium (Leica, Germany). Cryostat sections were cut at a thickness of 14 μm using a CryoStar NX50 device (Thermo Fisher Scientific, Germany). *In situ* hybridization (ISH) was performed using a QuantiGene ViewRNA Tissue Assay (Thermo Fisher Scientific, Germany) according to the manufacturer’s instructions and as previously described (Kallenborn-Gerhardt et al., 2017). Briefly, probes (all from Thermo Fisher Scientific) for *Hcn3* (diluted 1:40; catalog # VB1-19382, type 1 probe set and catalog # VB6-20079, type 6 probe set), *Rbfox3* (1:40; catalog # VB6-18012, type 6), *Slc17a8* (1:40; catalog # VB6-17592, type 6), *Ntrk2* (1:40; catalog # VB1-14047, type 1), *Ntrk3* (1:40; catalog # VB6-3200959, type 6), *Pvalb* (1:40; catalog # VB6-13220, type 6), *Kcnt1* (1:40; catalog # VB6-21049, type 6), calcitonin-related polypeptide alpha (*Calca*) (1:40; catalog # VB1-10936, type 1), *Trpm8* (1:40; catalog # VB6-18268, type 1) and scrambled control (1:40; catalog # VF1-17155, type 1 and catalog # VF6-18580, type 6) were incubated overnight at 40 °C (Thermobrite; Leica, Germany) followed by consecutive incubation with PreAmplifier Mix QT, Amplifier Mix QT, an alkaline phosphatase labeled probe against the Amplifier, AP Enhancer Solution, and Fast Red Substrate. Depending on the marker probes used, either the *Hcn3* type 1 or *Hcn3* type 6 probe set was used. Finally, the sections were mounted with Fluoromount G (Southern Biotech, United States). Images were taken using an Eclipse Ni-U microscope equipped with a monochrome DS-Qi2 camera (both from Nikon, Germany) and pseudocolored using NIS Elements software (Nikon, Germany).

### 2.4 Cell counting

For quantification of *Hcn3*-positive sensory neuron populations, 14-μm serial sections were prepared from lumbar (L4–L5) and thoracic DRGs (Th9–Th10) from 3 mice. Per animal, ≥3 DRG sections spaced 100 μm apart were counted manually (5,682 cells in total). Only cells showing clear staining above the background level, with a threshold set based on scrambled control hybridization, were included. The percentage of *Rbfox3-, Slc17a8*-, *Ntrk2*-, *Ntrk3*-, *Pvalb*-, *Kcnt1*-, *Calca*-, and *Trpm8*-positive neurons was expressed as a proportion of marker-positive cells per total number of *Hcn3*-positive neurons. For the calculation of the percentage of *Hcn3*-positive DRG neurons, the total number of DRG neuron somata was counted based on their autofluorescence visualized in the FITC channel.

### 2.5 RNA extraction and RT-qPCR

Mice were euthanized with CO_2_, and thoracic (Th9–Th10) DRGs were rapidly dissected, snap-frozen in liquid nitrogen, and stored at −80 °C. Total RNA was extracted under ribonuclease-free conditions using an innuPREP microRNA (miRNA) isolation kit (Analytik Jena; #C-6134, Germany). Reverse transcription was performed using a first-strand cDNA synthesis kit (Thermo Fisher Scientific, Germany) according to the manufacturer’s instructions. Quantitative real-time PCR (RT-qPCR) analysis was performed with a CFX96 Touch real-time system (Bio-Rad, Germany) using gene-specific primer pairs for murine *Hcn1* (fwd 5′-ctctttttgctaacgccgat-3′; rev 5′-cattgaaattgtccaccgaa-3′), *Hcn2* (fwd 5′-gtggagcgagctctactcgt-3′; rev 5′-gttcacaatctcctcacgca-3′), *Hcn3* (fwd 5′-ccgacggtcaacaagttctc-3′; rev 5′-cagcagcagcatgatgagat-3′), *Hcn4* (fwd 5′-gtacgcatcgtgaacctcattg-3′; rev 5′-tttcggcagttaaagttgatg-3′), and glyceraldehyde-3-phosphate dehydrogenase (*Gapdh*) (fwd 5′-caatgtgtccgtggatct-3′; rev 5′-gtcctcagtgtagcccaagatg-3′). To ensure specificity, reactions were performed in duplicate by incubating for 2 min at 50 °C and 15 min at 95 °C, followed by 40 cycles of 15 s at 95 °C and 1 min at 60 °C, including water controls. Reactions with Ct values >30 were excluded from the analysis because of reduced reliability at low template abundance. Relative *Hcn1, Hcn2, Hcn3,* and *Hcn4* expression levels were calculated using the comparative 2^-ΔΔCt^ method and normalized to those of *Gapdh* (Kallenborn-Gerhardt et al., 2017).

### 2.6 DRG neuron culture

Mice (4–8 weeks old) were euthanized by CO_2_ inhalation. Lumbar DRGs (L1-L5) and thoracic DRGs (Th1–Th12) were excised and transferred to HBSS (Thermo Fisher Scientific, United States). Following treatment with 2.5 U/mL dispase II and 500 U/mL collagenase IV (both from Roche, Switzerland) for 60 min, isolated cells were transferred onto coverslips coated with poly-d-lysine (250 μg/mL, Millipore, United States) and cultured in neurobasal medium supplemented with B27 (Thermo Fisher Scientific, United States), 100 μg/mL streptomycin, and penicillin (Roth, Germany) at 37 °C and 5% CO_2_. The cells were used for experiments within 24 h of plating.

### 2.7 Electrophysiological recordings

Whole-cell voltage-clamp recordings on cultured DRG neurons were performed at room temperature (20–22 °C) using a HEKA EPC 9 amplifier and Patchmaster software (HEKA Electronics, Germany). Offline analysis was performed using the Fitmaster software (HEKA Electronics, Germany) and GraphPad Prism 8. Micropipettes (3–5 MΩ) were pulled from borosilicate glass (Science Products, Germany) with a conventional micropipette puller (Model P-97, Sutter Instruments, United States). I_h_ was measured by continuous perfusion of the external solution with clamp steps of 4 s between −110 and −30 mV starting from a prepulse potential of −30 mV. The current densities were normalized to the cell capacitance (pA/pF). The pipette solution contained (mM): KCl 140, MgCl_2_ 2, EGTA 5, HEPES 10, and pH 7.4 adjusted with KOH. The external solution contained (mM): NaCl 140, KCl 5, CaCl_2_ 2, MgCl_2_ 2, HEPES 10, and pH 7.4 adjusted with NaOH. Evoked action potentials (APs) were elicited by 10-ms current injections starting at 0 pA in 20 pA increments to determine electrophysiological parameters. Thoracic or lumbar DRG neurons of small and medium size (<30 μm, ≤35 pF) were chosen for electrophysiological recordings.

### 2.8 Statistical analysis

GraphPad Prism 8 software was used for statistical analysis. No sample size calculations were performed; however, the sample sizes employed in this study are similar to those used in the field (Hussein et al., 2019; Miyahara et al., 2021; Stieglitz et al., 2017). The treatment groups were randomized and evenly distributed across cages and sexes. The Kolmogorov–Smirnov test was used to assess the normal distribution of data within groups. Normally distributed data were analyzed using an unpaired *t*-test or repeated measures analysis of variance (ANOVA) with a Bonferroni *post-hoc* test for multiple comparisons. In the cotton swab test on hairy skin, the proportion of each response category was compared between genotypes using the chi-squared test. A probability value *p* < 0.05 was considered significant. All data are presented as mean ± SEM.

## 3 Results

### 3.1 Expression of HCN3 in molecularly defined subsets of sensory neurons

**Figure 1:**
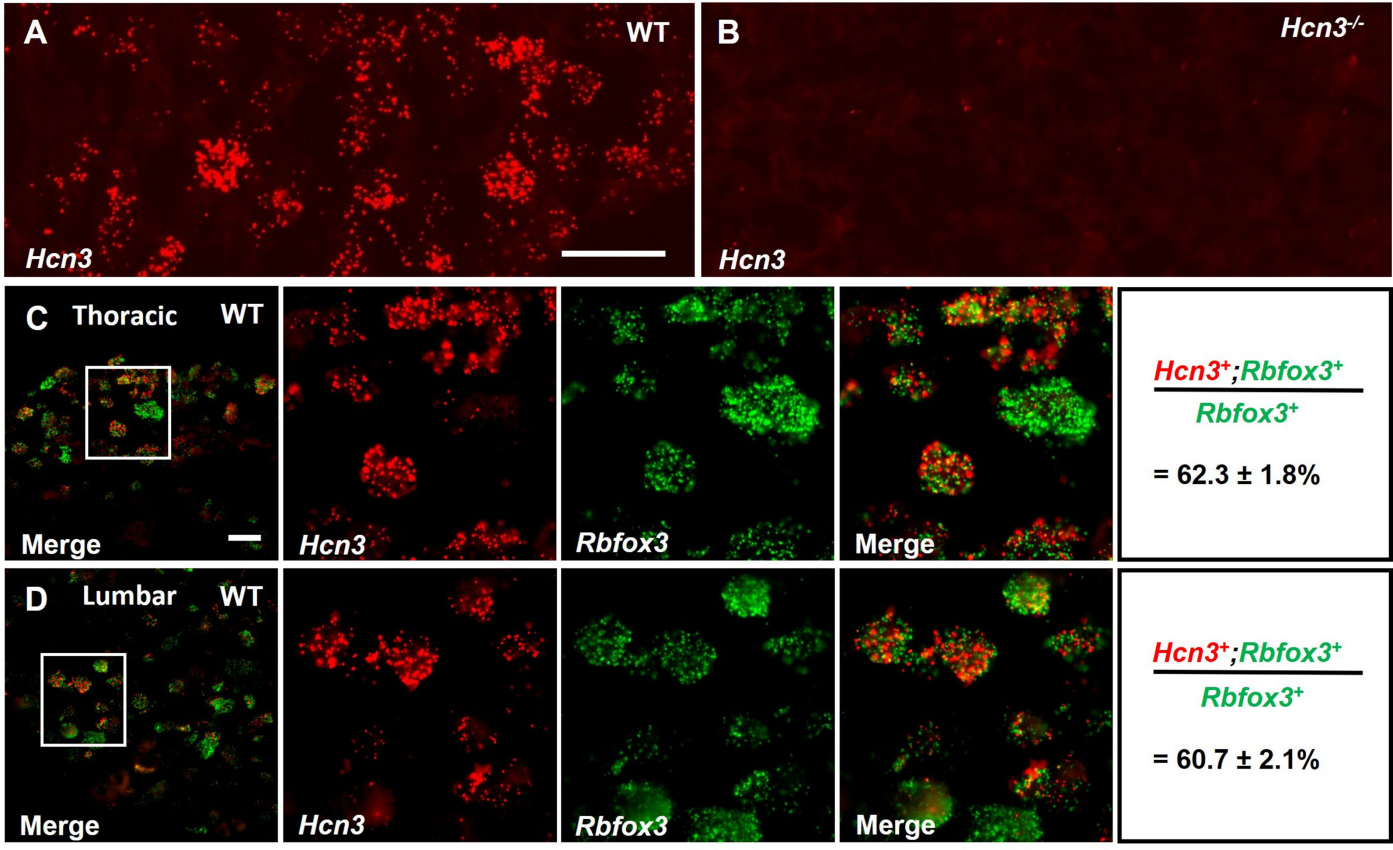
Distribution of *Hcn3* mRNA in dorsal root ganglia (DRG) of mice. **(A)** Fluorescent *in situ* hybridization detected *Hcn3* mRNA in thoracic DRGs of WT mice. **(B)** No hybridization signal was detected in the DRGs of *Hcn3^−/−^* mice. **(C,D)** Double *in situ* hybridization with the pan-neuronal marker *Rbfox3* in the thoracic **(C)** and lumbar **(D)** DRGs of WT mice. A quantitative summary of *Rbfox3*-positive neurons expressing *Hcn3* is presented on the right (*n* = 3 animals). Scale bar: 25 μm **(A, C)**. Raw numbers of the double *in situ* hybridization experiments are presented in Supplementary Tables S1, S2.

We first investigated the cellular distribution of *Hcn3* mRNA in DRGs using fluorescent *in situ* hybridization. In preparation for the behavioral and electrophysiological experiments of this study, we analyzed the *Hcn3* distribution in thoracic (Th9–Th10) DRGs, which innervate hairy skin, and lumbar (L4–L5) DRGs, which innervate both glabrous and hairy skin (Boada et al., 2010), because the gene expression pattern can vary depending on the segmental location of the DRG (Chaplan et al., 2003; Liu et al., 2007; Vandewauw et al., 2013). We detected abundant hybridization signals of *Hcn3* in the thoracic DRGs of WT mice (Figure 1A), whereas no signal was detected in DRGs from *Hcn3^−/−^* mice (Figure 1B), confirming the specificity of the *in situ* hybridization probes. A control experiment using RT-qPCR confirmed the deletion of *Hcn3* in the thoracic DRGs of *Hcn3^−/−^* mice and revealed that the mRNA levels of *Hcn1, Hcn2,* and *Hcn4* were unaltered between genotypes (Supplementary Figure S1), suggesting that *Hcn3* deficiency did not result in compensatory regulation of other HCN channels. Double *in situ* hybridization with the pan-neuronal marker *Rbfox3* (which codes for the “neuronal nuclei” antigen NeuN) revealed that 62.3 ± 1.8% of *Rbfox3*-positive thoracic DRG neurons express *Hcn3* (Figure 1C), whereas *Hcn3* was detected in 60.7 ± 2.1% of *Rbfox3*-positive lumbar DRG neurons (Figure 1D), suggesting that the gross *Hcn3* distribution is similar in thoracic and lumbar DRGs. The percentage of *Hcn3*-expressing DRG neurons largely corresponds to a previous study, in which 61.0 ± 1.0% of lumbar DRG neurons in mice were found to be immunoreactive for HCN3 protein (Lainez et al., 2019).

**Figure 2:**
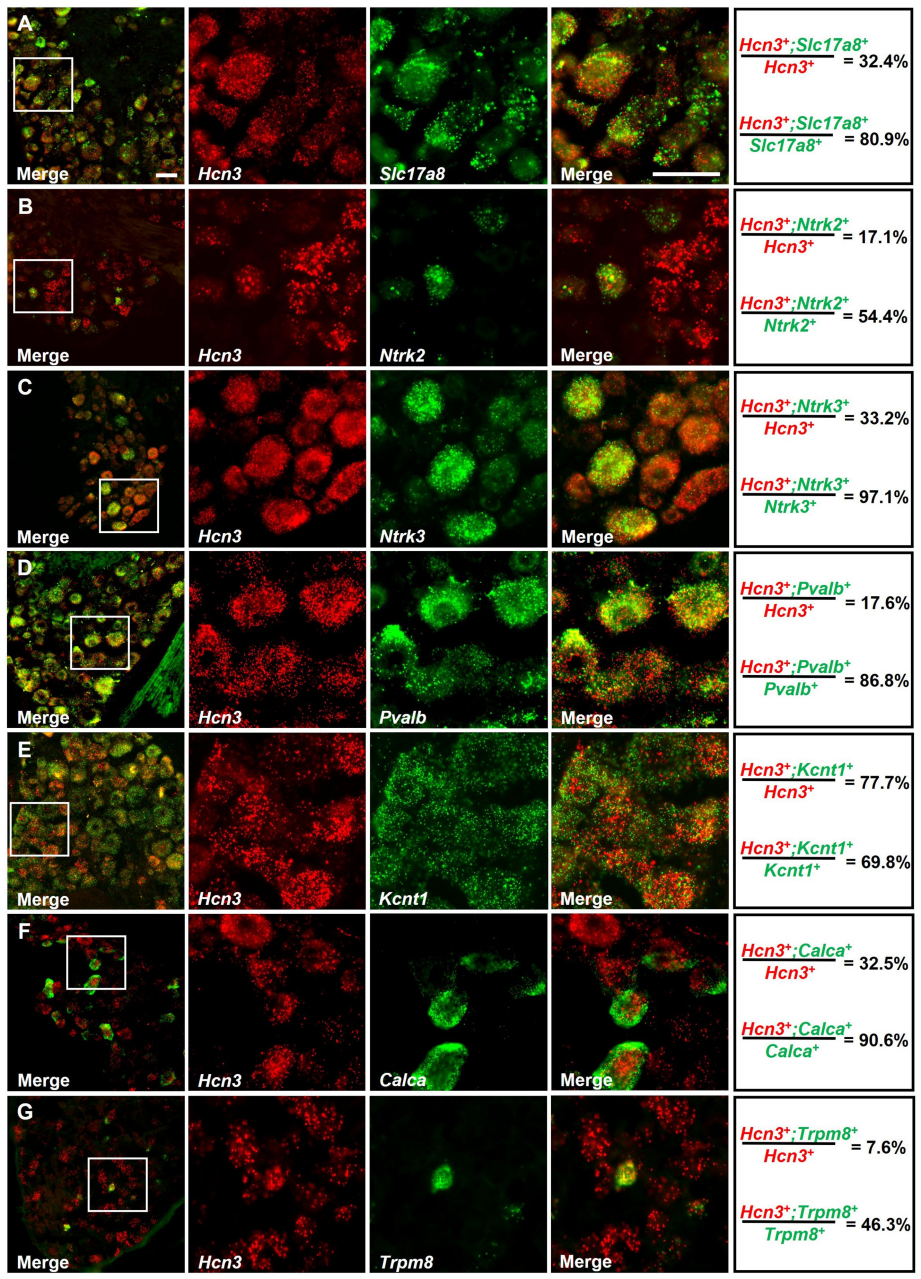
Double *in situ* hybridization for *Hcn3* and established markers in thoracic DRGs. *Hcn3* mRNA was detected in neurons positive for **(A)** *Slc17a8*, a marker of C-LTMR; **(B)** *Ntrk2*, a marker of Aδ-LTMR; **(C)** *Ntrk3*, a marker of A-beta SA1-LTMR and A-beta Field-LTMR; and **(D)** *Pvalb*, which labels proprioceptors. In nociceptive sensory neurons, *Hcn3* was co-expressed in cells positive for **(E)** *Kcnt1*, a marker of non-peptidergic C-fiber nociceptors; **(F)** *Calca*, which marks peptidergic nociceptors; and **(G)** *Trpm8*, a marker of cold-sensitive peptidergic nociceptors. A quantitative summary is presented on the right (*n* = 3 animals). Scale bar: 25 μm **(A)**. Raw numbers of the double *in situ* hybridization experiments are presented in Supplementary Table S1.

**Figure 3:**
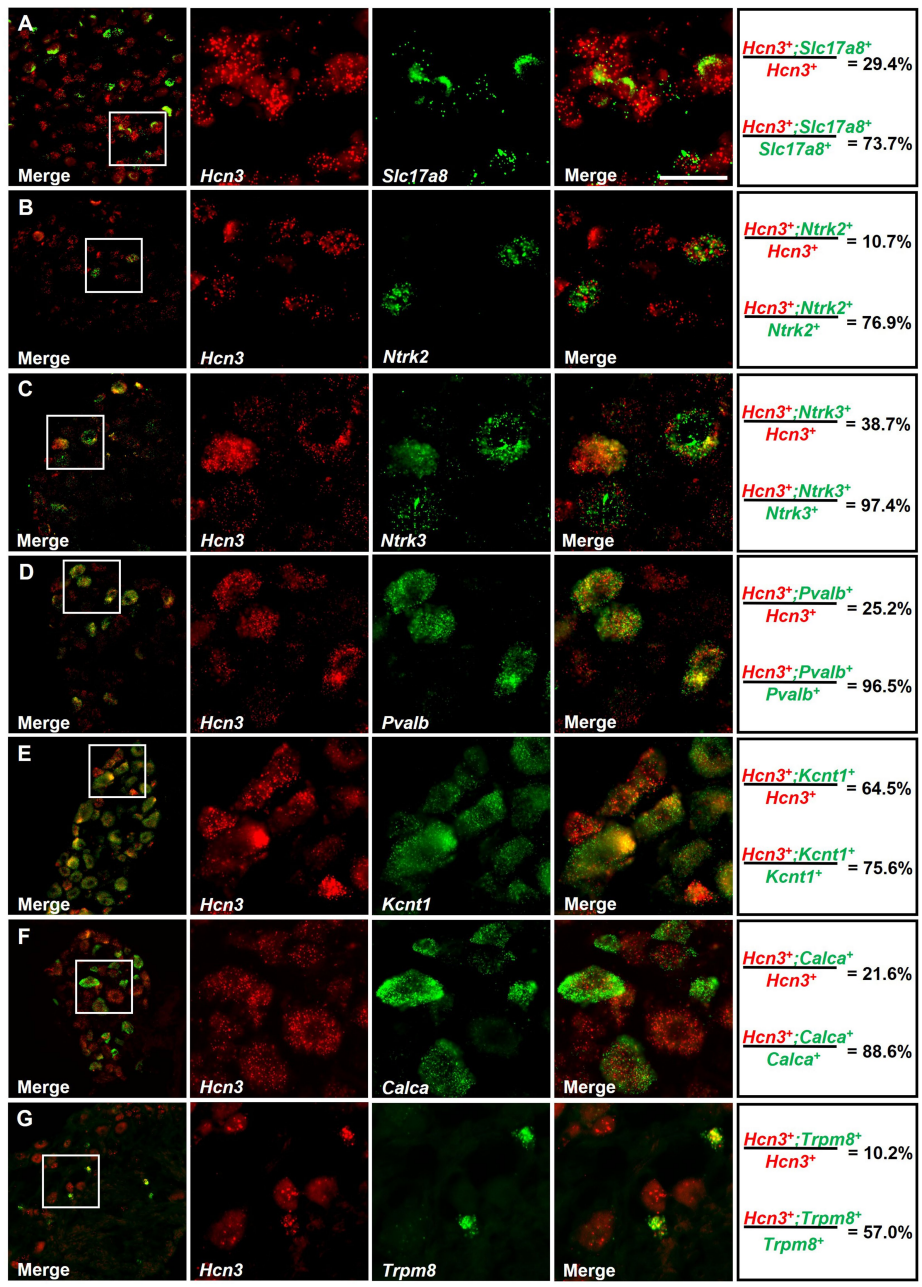
Double *in situ* hybridization for *Hcn3* and established markers in lumbar DRGs. *Hcn3* mRNA was detected in neurons positive for **(A)** *Slc17a8*, a marker of C-LTMR; **(B)** *Ntrk2*, a marker of Aδ-LTMR; **(C)** *Ntrk3*, a marker of A-beta SA1-LTMR and A-beta Field-LTMR; and **(D)** *Pvalb*, which labels proprioceptors. In nociceptive sensory neurons, *Hcn3* was co-expressed in neurons positive for **(E)** *Kcnt1*, a marker of non-peptidergic C-fiber nociceptors; **(F)** *Calca*, which marks peptidergic nociceptors; and **(G)** *Trpm8*, a marker of cold-sensitive peptidergic nociceptors. A quantitative summary is presented on the right (*n = 3* animals). Scale bar: 25 m **(A)**. Raw numbers of the double *in situ* hybridization experiments are presented in Supplementary Table S2.

To analyze the cellular distribution of *Hcn3* in DRG neuron subpopulations in more detail, we performed double *in situ* hybridization experiments using different cellular markers (Figures 2, 3). In thoracic DRGs, *Hcn3* was detected in 80.9 ± 6.3% of neurons positive for *Slc17a8* (Vglut3), a marker of C-LTMR (Li et al., 2011), and in 54.4 ± 1.5% of neurons expressing *Ntrk2* (TrkB), a marker of Aδ-LTMR (Li et al., 2011; Figures 2A,B). Furthermore, *Hcn3* was present in 97.1 ± 1.8% of cells positive for *Ntrk3* (TrkC), a marker of A-beta SA1-LTMR and A-beta Field-LTMR (Zheng et al., 2019), and in 86.8 ± 7.7% of neurons expressing *Pvalb* (Zheng et al., 2019), which labels proprioceptors (Figures 2C,D). In nociceptive sensory neurons, *Hcn3* was co-expressed in 69.8 ± 5.4% of cells positive for *Kcnt1*, a marker of non-peptidergic C-fiber nociceptors (Lu et al., 2015); in 90.6 ± 2.4% of cells expressing *Calca,* which encodes calcitonin gene-related peptide (CGRP), a marker of peptidergic nociceptors (Basbaum et al., 2009); and in 46.3 ± 2.8% of cells positive for *Trpm8*, a marker of cold-sensitive peptidergic nociceptors (Dhaka et al., 2007; Figures 2E–G). A similar distribution pattern was observed in lumbar DRGs: *Hcn3* was detected in 73.7 ± 5.4% of neurons positive for *Slc17a8*, 76.9 ± 7.9% of neurons expressing *Ntrk2*, 97.4 ± 1.7% of *Ntrk3*-positive cells, and 96.5 ± 2.0% of neurons positive for *Pvalb* (Figures 3A–D). Moreover, *Hcn3* was seen in 75.6 ± 10.3%, 88.6 ± 5.9%, and 57.0 ± 7.7% of lumbar DRG neurons positive for *Kcnt1*, *Calca*, and *Trpm8*, respectively (Figures 3E–G). Together, these data suggest that *Hcn3* is expressed in all assessed populations of DRG neurons, including Aβ-LTMRs, Aδ-LTMRs, C-LTMRs, and nociceptors.

### 3.2 HCN3 contributes to touch sensation on hairy skin

**Figure 4:**
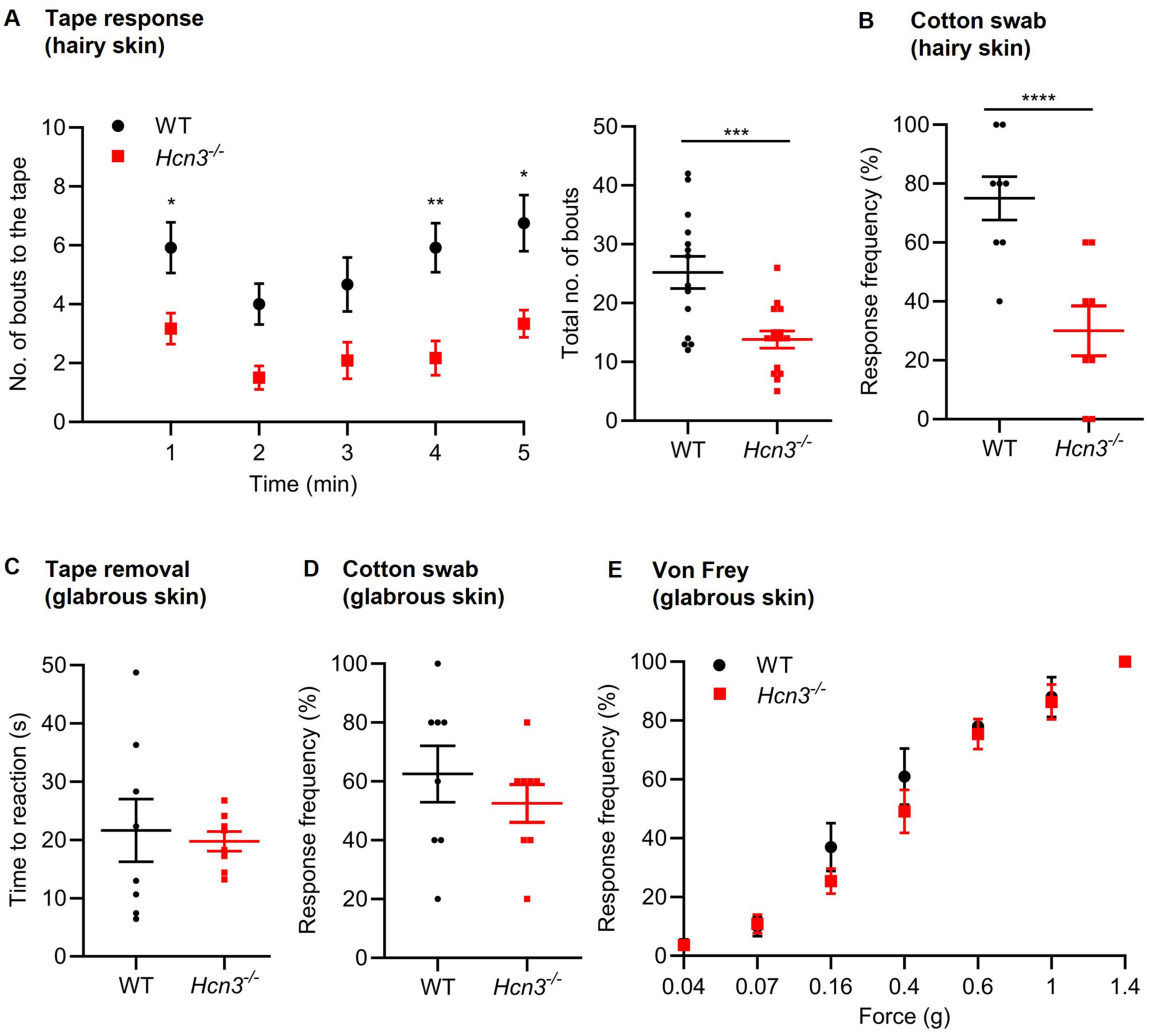
Touch sensation tests in *Hcn3^−/−^* mice. **(A)** In the tape response test, an adhesive tape was placed on the hairy skin of the back. *Hcn3^−/−^* mice (*n* = 12) exhibited a reduced number of bouts to the tape during a 5 min observation period compared to WT mice (*n* = 10). The time course is shown on the left, and the sum of the reactions is shown on the right. ^*^p < 0.05, **p < 0.01, ***p < 0.001, repeated measures analysis of variance (ANOVA) with Bonferroni multiple testing (time course) or unpaired *t*-test (sum: *p* = 0.0009; 95% CI, −17.69 to −5.14). **(B)** In the cotton swab test on hairy skin, the dorsal fur was stroked with a “puffed” cotton swab (five applications). The frequency of unpleasant reactions was significantly reduced in *Hcn3^−/−^* mice (*n* = 8) compared to that in WT mice (*n* = 8). ****p < 0.0001 (exact *p*-value not given in Prism), Chi-squared test. **(C)** In the tape removal test, cutaneous mechanosensitivity on glabrous skin was measured by applying an adhesive tape to the plantar area of the hindpaw. *Hcn3^−/−^* and WT mice (*n* = 8 per group; *p* = 0.74; 95% CI, −10.21 to 14 s) showed similar reaction times. **(D)** Hindpaw withdrawal frequencies in response to a cotton swab stroke onto glabrous skin (5 applications) were similar between *Hcn3^−/−^* mice and WT mice (*n* = 8 per group; *p* = 0.95, Chi-squared test). **(E)** Hindpaw withdrawal responses elicited by applying a range of von Frey filaments (10 applications per filament) onto glabrous skin were similar in HCN3^−/−^ (*n* = 11) and WT mice (*n* = 10). Data are presented as mean ± SEM **(A,C,E)** or as median with interquartile range **(B,D)**.

We next explored the behavior of *Hcn3^−/−^* and WT littermates in models that sense touch, heat, and cold. To test their response to innocuous mechanical stimuli, we applied an adhesive tape to the hairy skin of the back. Interestingly, *Hcn3^−/−^* mice exhibited a considerably reduced number of bouts directed toward the tape during a 5-min observation period (Figure 4A). In a second test on hairy skin, we used a “puffed” cotton swab and stroked the dorsal fur from rostral to caudal for <1 s, repeating this 5 times. Using this light-touch stroke assay, we observed a significantly reduced number of unpleasant reactions in *Hcn3^−/−^* mice (Figure 4B). These data suggest that HCN3 contributes to the sensation of touch on the hairy skin. We then assessed cutaneous mechanosensitivity on glabrous skin by applying an adhesive tape to the plantar area of the hindpaw. *Hcn3^−/−^* and WT mice showed similar reaction times to tape removal in this test (Figure 4C), in contrast to the profound differences observed in the tape response test on hairy skin. Similarly, hindpaw withdrawal responses elicited by stroking glabrous skin with a “puffed” cotton swab or applying a range of von Frey filaments onto glabrous skin were similar between genotypes (Figures 4D,E). Together, these data implicate a function of HCN3 in the sensation of mechanical stimuli on hairy skin, but not on glabrous skin.

**Figure 5:**
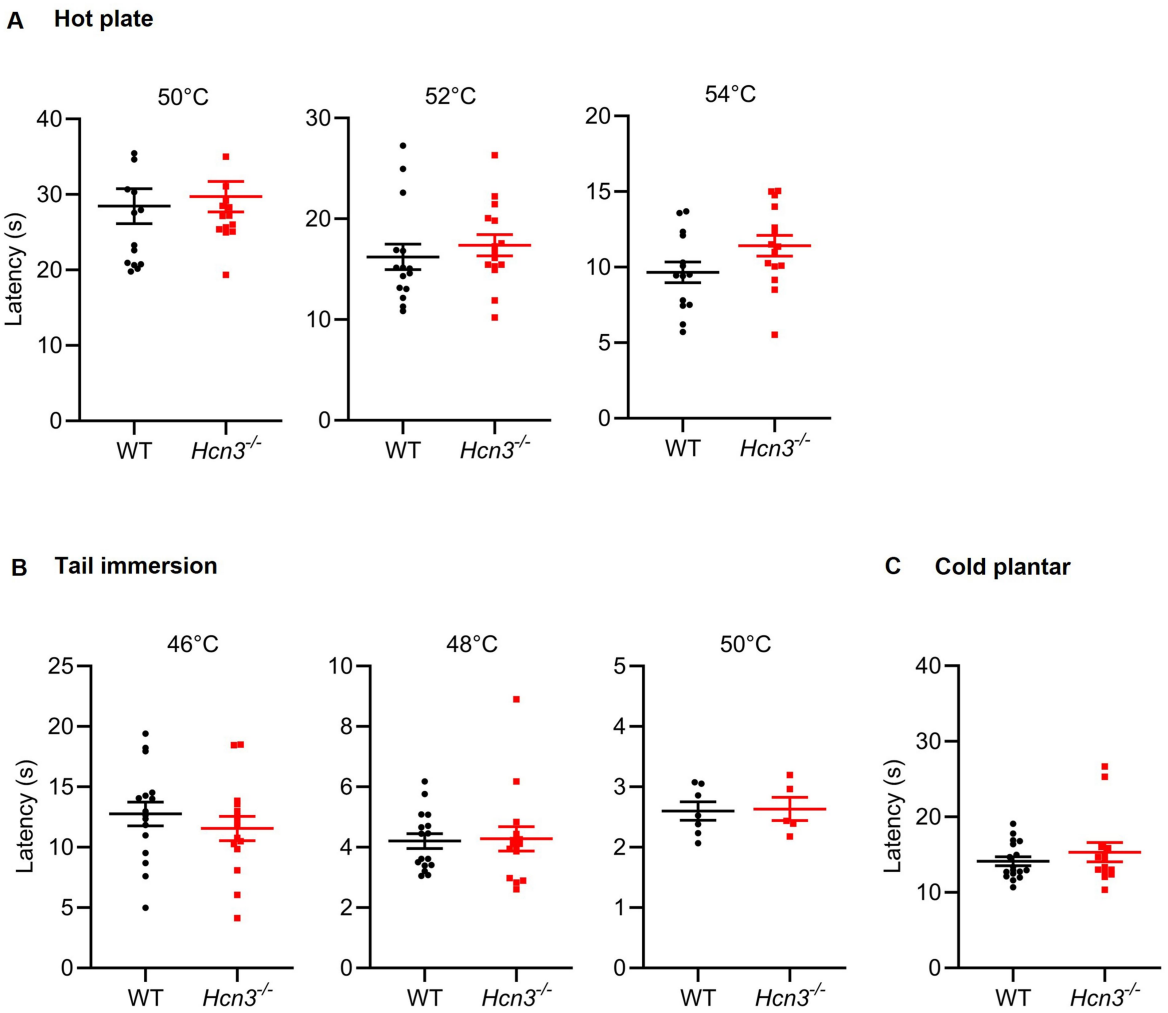
Acute nociceptive tests in *Hcn3^−/−^* mice. **(A)** *Hcn3^−/−^* and WT mice exhibited similar latencies on a hot plate set at 50 °C (*n* = 15; *p* = 0.68; 95% CI: −5.03 to 7.53 s), 52 °C (*n* = 15; *p* = 0.49; 95% CI: −2.22 to 4.55 s), or 54 °C (*n* = 15; *p* = 0.082; 95% CI: −0.24 to 3.76 s). **(B)** *Hcn3^−/−^* mice showed normal responses to noxious heat stimuli in the tail immersion test at 46 °C (*n* = 15; *p* = 0.22; 95% CI: −2.43 to 9.95 s), 48 °C (*n* = 15; *p* = 0.14; 95% CI: −0.27 to 1.77 s), or 50 °C (*n* = 7; *p* = 0.76; 95% CI: −0.69 to 0.52 s), compared to WT mice. **(C)** The reaction time to a cold stimulus applied to the hindpaw in the cold plantar test was similar between *Hcn3^−/−^* mice (*n* = 14) and WT mice (*n* = 17; *p* = 0.38; 95% CI: −3.9 to 1.53 s). Data are presented as mean ± SEM.

We then characterized the behavior of *Hcn3^−/−^* mice and WT littermates in models of acute nociceptive pain. We found that *Hcn3^−/−^* and WT mice exhibited similar latencies on a hot plate set at 50, 52, or 54 °C (Figure 5A). *Hcn3^−/−^* mice also showed normal responses to noxious heat stimuli in the tail immersion test at 46, 48, or 50 °C (Figure 5B). Furthermore, the reaction time to a cold stimulus applied to the hindpaw in the cold plantar test was similar between genotypes (Figure 5C). The unaltered nociceptive behavior of *Hcn3^−/−^* mice is in line with a previous study that reported that mechanical withdrawal thresholds after hindpaw stimulation with a Dynamic Plantar Aesthesiometer, responses to paw pressure in the Randall-Sellito test, paw withdrawals following a sharp pinprick, and withdrawal thresholds of the hindpaw after applying thermal stimuli in the Hargreaves test were unaffected in *Hcn3^−/−^* mice (Lainez et al., 2019). Together, these data suggest that HCN3 is dispensable for the sensation of noxious mechanical or thermal stimuli applied to the hindpaw.

### 3.3 HCN3 contributes to I_h_ in thoracic but not lumbar DRG neurons

**Figure 6:**
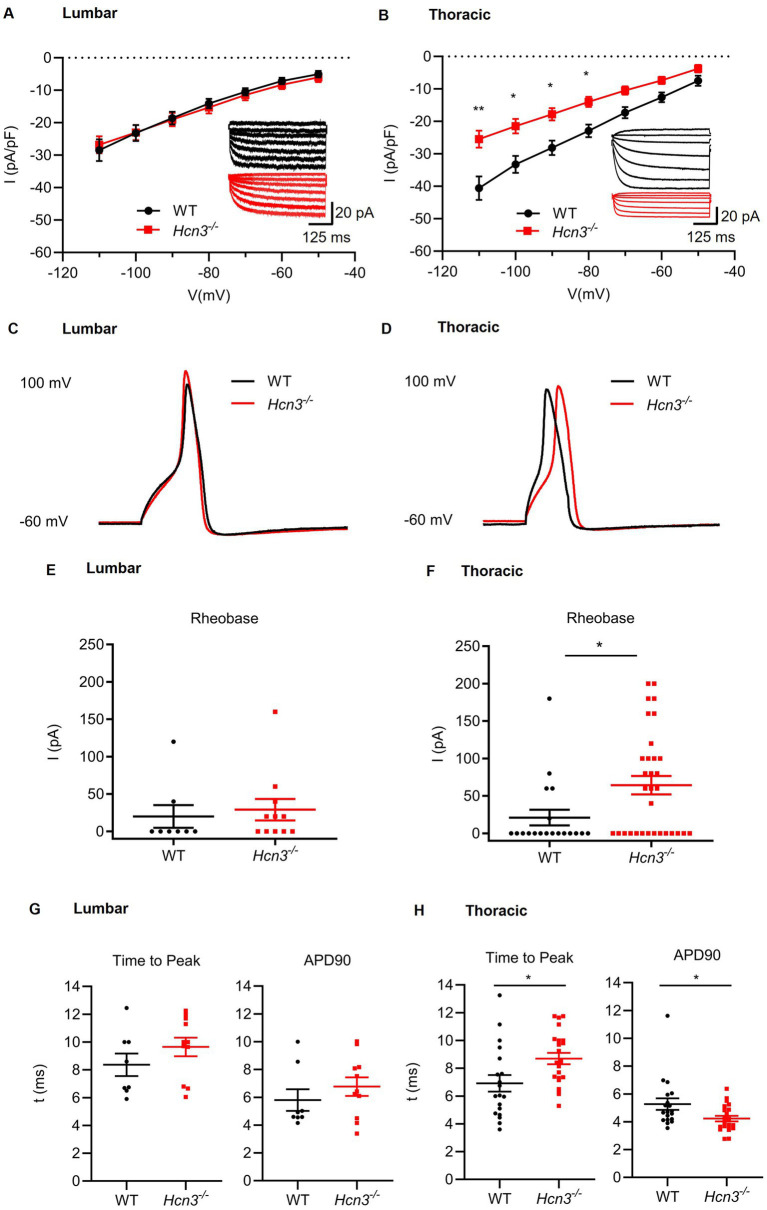
Whole-cell patch-clamp recordings in thoracic and lumbar DRG neurons of *Hcn3^−/−^* and WT mice. **(A,B)** Hyperpolarizing voltage steps from −30 mV to −110 mV were applied to measure the inward current, I_h_. **(A)** No differences in current densities were seen in lumbar *Hcn3^−/−^* neurons (*n* = 33 neurons from 5 mice) compared to lumbar WT neurons (*n* = 27 neurons from 5 mice), which is also shown in the original registration of I_h_ *(inset)*. **(B)** The current densities of thoracic *Hcn3^−/−^* neurons were significantly altered (−25.5 ± 2.6 pA/pF at −110 mV; *n* = 40 neurons from 4 mice) compared to the current densities of thoracic WT neurons (−40.6 ± 3.6 pA/pF at −110 mV; *n* = 36 neurons from 6 mice), which is also shown in an original registration of I_h_ *(inset)*. ^*^p < 0.05, **p < 0.01, repeated measures analysis of variance (ANOVA) with Bonferroni multiple testing. **(C–H)** Evoked action potentials (APs) in lumbar (WT: *n* = 8 neurons from 2 mice; *Hcn3^−/−^*: *n* = 11 neurons from 2 mice) and thoracic DRG neurons (WT: *n* = 19 neurons from 5 mice; *Hcn3^−/−^*: *n* = 22 neurons from 4 mice) were elicited by 10 ms current injections starting at 0 pA in 20 pA steps. **(C,D)** Representative evoked APs at rheobase current injections in lumbar and thoracic DRG neurons. **(E,F)** The mean rheobase (minimum amplitude of current required to generate an AP) in lumbar DRG neurons (*p* = 0.35) was similar between genotypes, while the mean rheobase in thoracic DRG neurons (*p* = 0.017) was significantly higher in *Hcn3^−/−^* mice than in WT mice (unpaired *t*-test). **(G)** In lumbar DRG neurons, the time to peak and the AP duration at 90% repolarization (APD90) were similar between genotypes. **(H)** In thoracic DRG neurons, the time to peak (*p* = 0.016; 95% CI: −3.21 to −0.34 ms) and the APD90 (*p* = 0.02; 95% CI: 0.15–1.9 ms) were significantly altered in *Hcn3^−/−^* mice compared to WT mice, whereas the resting membrane potential (RMP) and the amplitude of the measured APs were unaltered. *p < 0.05, unpaired *t*-test. Data are presented as mean ± SEM or as median with interquartile range **(G,H)**.

Using whole-cell patch-clamp recordings, we investigated how the inward current mediated by HCN channels, known as I_h_, is affected *by Hcn3* deficiency in the thoracic (Th9–Th10) and lumbar (L4–L5) DRG neurons. For the measurements, DRG neurons of small and medium sizes were chosen. We applied a hyperpolarizing voltage step from −30 mV, at which all HCN isoforms are deactivated, to −110 mV, which achieved activation of the HCN channels and the maximum inward current I_h_ (Figures 6A,B). No differences in current densities were observed in lumbar *Hcn3^−/−^* neurons compared to lumbar WT neurons (Figure 6A). In contrast, the current densities of thoracic *Hcn3^−/−^* neurons were significantly altered (−25.5 ± 2.6 pA/pF at −110 mV) compared to thoracic WT neurons (−40.6 ± 3.6 pA/pF at −110 mV) (Figure 6B). These data suggest that HCN3 contributes to I_h_ in thoracic DRG neurons but not in lumbar DRG neurons.

We next analyzed evoked action potentials (APs), which were elicited by 10-ms current injections starting at 0 pA in 20 pA steps. In these recordings, some cells elicited APs with 0 pA injection, whereas other cells elicited APs after current injection above 0 pA. Representative APs evoked during rheobase current injections in lumbar and thoracic DRG neurons are shown in Figures 6C,D, respectively. In lumbar DRG neurons, the mean rheobase (minimum amplitude of current required to generate an AP) was similar in *Hcn3^−/−^* and WT mice (Figure 6E). In contrast, in thoracic DRG neurons, the mean rheobase level was significantly higher in *Hcn3^−/−^ mice* than in WT mice (Figure 6F). In lumbar DRG neurons, the time to peak and AP duration at 90% repolarization (APD90) were similar between the groups (Figure 6G). In thoracic DRG neurons, however, the time to peak was significantly increased in *Hcn3^−/−^* mice, while APD90 was significantly reduced in *Hcn3^−/−^* mice (Figure 6H). In contrast, the resting membrane potential (RMP) and amplitude of the measured APs were not affected by *Hcn3* deletion in the lumbar and thoracic DRG neurons (data not shown). These data support the contribution of HCN3 to I_h_ and APs in thoracic sensory neurons and suggest that the functional role of HCN3 may vary depending on the segmental location of the DRG.

## 4 Discussion

Here, we identify HCN3 as a contributor to the light touch sensation on hairy skin. We demonstrate that *Hcn3* mRNA is expressed in a variety of sensory neurons, including populations that are important for touch sensation. Behavioral analyses revealed that *Hcn3^−/−^* mice show reduced responses to mechanical stimuli on hairy skin, while perception on glabrous skin was preserved. Moreover, patch-clamp recordings indicate that HCN3 significantly contributes to I_h_ current density and AP kinetics in thoracic (Th9–Th10) but not in lumbar (L4–L5) DRG neurons. Notably, these functional differences were present despite comparable *Hcn3* expression in thoracic and lumbar DRGs.

The perception of innocuous touch sensations relies on specialized somatosensory LTMRs. Previous studies have identified five principal LTMRs: C-LTMRs, Aδ-LTMRs, Aβ RA-LTMRs, Aβ SAI-LTMRs, and Aβ Field-LTMRs (Bai et al., 2015; Handler and Ginty, 2021; Zimmerman et al., 2014). These neuronal populations react to innocuous mechanical stimulation acting on the hairy and/or glabrous skin (Rutlin et al., 2014). C-LTMRs exclusively innervate the hairy skin as longitudinal lanceolate nerve endings (Li et al., 2011; Zimmerman et al., 2014) and normally convey innocuous mechanical (hair deflection and light touch) and cooling sensations. In addition, C-LTMRs are involved in processing gentle and affective touch (Löken et al., 2009). Aδ-LTMRs, or D-hair cells, also exclusively innervate hairy skin and are maximally excited by gentle touch (Li et al., 2011). The polarized organization of (TrkB^+^) Aδ-LTMR lanceolate endings along just one side of hair follicles renders the Aδ-LTMRs uniquely direction sensitive, with the strongest responses evoked by deflection of awl, auchene, or zigzag hairs of trunk skin along the caudal-to-rostral axis (Rutlin et al., 2014) and to the deflection of hairs on the ventral paw along the rostral-to-caudal axis (Handler and Ginty, 2021; Walcher et al., 2018). The hairy skin-innervating Aβ RA-LTMRs also form longitudinal lanceolate endings that envelope certain hair follicle types, whereas hairy skin-innervating Aβ SAI-LTMRs are associated with large groups of Merkel cells that form crescent-shaped touch domes (Handler and Ginty, 2021). Furthermore, hairy skin innervating Aβ Field-LTMRs forms circumferential endings that wrap around hair follicles, similar to corkscrews (Bai et al., 2015; Handler and Ginty, 2021). Although typically activated by static indentation only at high forces, Aβ Field-LTMRs are sensitive to gentle strokes across the skin (Handler and Ginty, 2021). This array of mechanosensory structures converts innocuous forces acting on hairy skin into electrical signals that propagate to the spinal cord and brain.

However, how a stimulus is detected and converted into electrical signals on hairy skin remains poorly understood. Putative mechanosensors in hair follicle-associated LMTRs include the acid-sensing Na^+^ channel ASIC2 (Roza et al., 2004), TRPV4 (Suzuki et al., 2003), members of the KCNK subfamily (Noël et al., 2009), KCNQ4 (Heidenreich et al., 2011), and, in particular, Piezo2 (Ranade et al., 2014). Our study suggests that HCN3 is one of the channels that contribute to the processing of electrical signals in sensory neurons after applying innocuous touch stimuli onto hairy skin. In general, the activation of an HCN channel generates an inward current and depolarizes the RMP to initiate spontaneous firing, i.e., it acts as a pacemaker (He et al., 2014). Accordingly, previous electrophysiological studies have revealed that inward currents mediated by HCN channels influence the excitability of sensory neurons (Gao et al., 2012). Although speculative, it might be possible that a deletion of HCN3 leads to inefficient hyperpolarization and reduced excitability of sensory neurons after initiation of touch perception by, e.g., Piezo2 in LTMRs. The functional contribution of HCN channels to innocuous touch sensations was demonstrated in earlier studies that investigated tactile-related phenotypes in mouse models and patients with autism spectrum disorder (ASD), a neurodevelopmental disorder characterized by impairments in social communication and interactions (Orefice et al., 2019). Interestingly, *Hcn1*-deficient mice exhibited enhanced hairy skin sensitivity and deficits in texture discrimination (Orefice et al., 2019). Furthermore, reduced I_h_ together with enhanced neuronal excitability was measured in human neurons with ASD-associated mutations (Yi et al., 2016). In our electrophysiological studies, we detected a remaining fraction of 62% I_h_ density in the sensory neurons of *Hcn3^−/−^* mice. As *Hcn4* is virtually not expressed in sensory neurons (Chaplan et al., 2003; Supplementary Figure S1), it is likely that the remaining I_h_ in *Hcn3^−/−^* mice is generated by HCN1 and/or HCN2, which are localized in distinct populations of sensory neurons (Dini et al., 2018; Jiang et al., 2008; Kouranova et al., 2008). Moreover, our evoked AP recordings revealed significant differences between *Hcn3^−/−^* and WT neurons in the time to peak, APD90, and rheobase. Our data suggest that in *Hcn3^−/−^* neurons, more current is required to charge the cell membrane and, therefore, reach the threshold potential for an AP. This could also change the availability of voltage-gated Ca ^2+^- and Na^+^ channels, thereby leading to significant changes in time to peak and the action potential duration (APD90). Similarly, a previous study demonstrated that a lack of I_h_ may lead to a shortening of AP duration (Fenske et al., 2011). These findings led us to speculate that HCN3 is involved in stabilizing the resting membrane potential and acts as a functional antagonist of hyperpolarizing K^+^ currents during late repolarization. Overall, this reflects the complex neuronal functions of HCN3 that have been reported in earlier studies. For example, Stieglitz et al. (2017) found that HCN3 channels are expressed in the basolateral amygdala and mouse brain and that a knockout leads to an impairment in fear extinction and an increase in fear generalization. Another study reported that GABA_A_ receptors anchor HCN2/ HCN3 subunits in dopaminergic neurons and that the lack of this complex leads to prolonged inhibition of neuronal firing as well as increased anxiety-like behavior in response to stress (Pérez-Garci et al., 2025).

We can only speculate about the reasons for the different functions of HCN3 in thoracic and lumbar DRG neurons. In addition to the general differences in signal transduction in neurons innervating the hairy or glabrous skin, other factors that are present differently depending on the segmental location of the DRG could play a role. For example, specific neuronal networks via cAMP regulation (Mu et al., 2023; Wang et al., 2007) or specific interactions with other ion channels, such as Slack (Wu et al., 2024), might be different in the thoracic and lumbar DRG. It could also be possible that the auxiliary subunit tetratricopeptide repeat-containing Rab8b-interacting protein (TRIP8b), which interacts with HCN channels by regulating surface trafficking and I_h_ density (Han et al., 2020; Lewis et al., 2011), has different functions in the thoracic and lumbar DRG. Another reason could be the DRG segment-specific formation of heteromeric channels. Previous studies have shown that HCN1 and HCN2 can form heteromers with unique channel characteristics (Ulens and Tytgat, 2001).

In line with our finding that deletion of HCN3 affects I_h_ and APs in sensory neurons that innervate the LTMRs of hairy skin, it would be interesting to study how pharmacological modulation of HCN3 affects the excitability of these neurons. However, to the best of our knowledge, specific blockers or stimulators of HCN3 channels are not yet available. Furthermore, from a translational perspective, it would be important to investigate whether HCN3 is also expressed in human sensory neurons and functionally involved in touch sensation on hairy skin.

In summary, the results of this study show that HCN3 plays a functional role in the tactile perception of hairy skin, whereas its function in the perception of glabrous skin or nociception is negligible. The segmental specificity of electrophysiological effects highlights the need for further studies to clarify the mechanisms that determine the different functions of HCN3 in populations of sensory neurons. Additionally, a detailed analysis of the interactions of HCN3 with other ion channels and regulatory proteins could provide valuable insights into the complex modulation of sensory signaling pathways.

## Data Availability

The raw data supporting the conclusions of this article will be made available by the authors, without undue reservation.
