# Supplementary material for: Impaired touch sensation on hairy skin in HCN3-deficient mice
Source: Front Neurosci. 2026 Jan 12;19:1697582. doi: 10.3389/fnins.2025.1697582 (PMC12834049; doi:10.3389/fnins.2025.1697582)
Supplement: Supplementary file 1 [file Data_Sheet_1.docx]

Supplementary Material

**
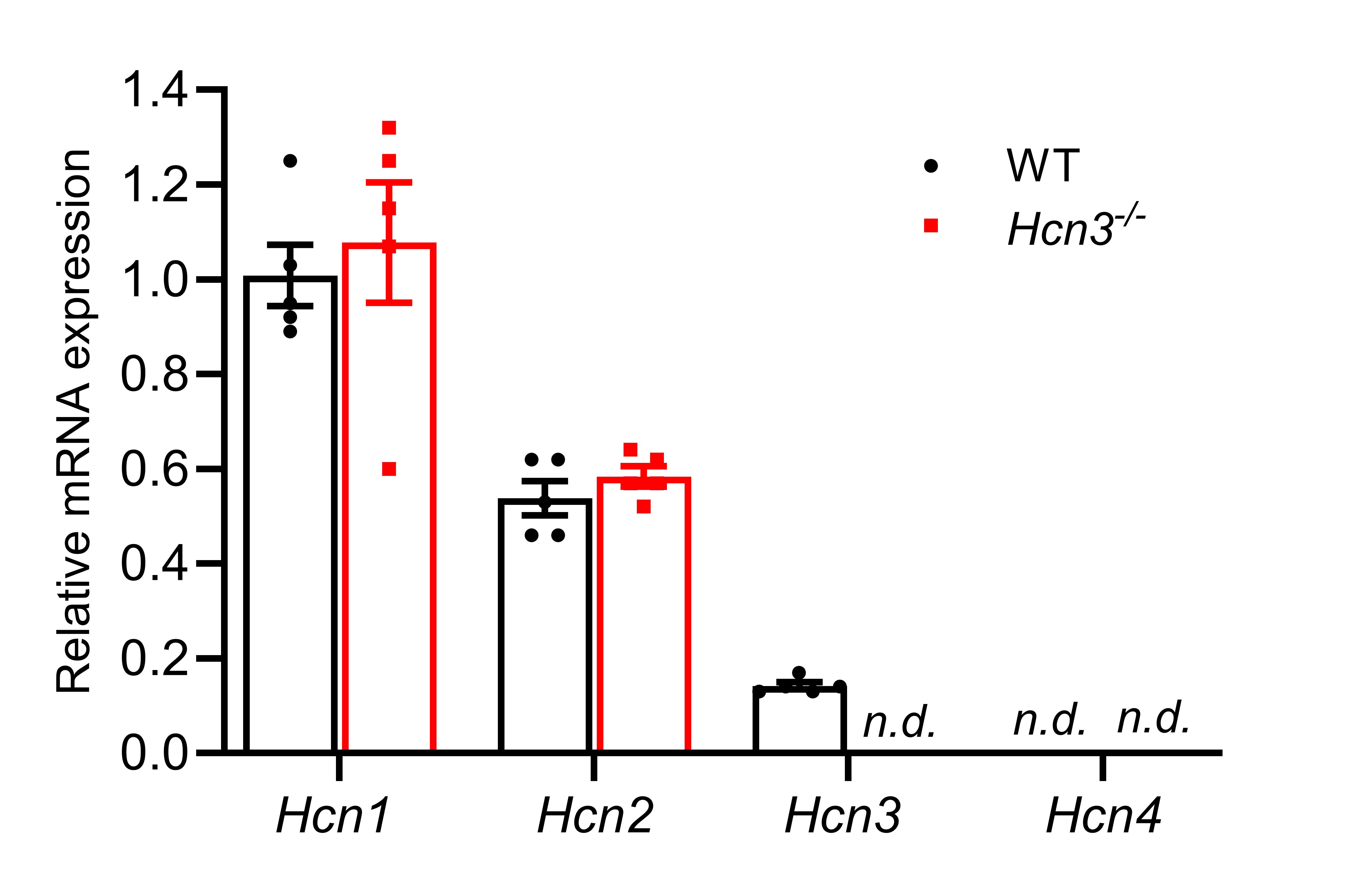
**

**Supplementary Figure 1.** Quantitative real-time RT-PCR analysis of *Hcn1, Hcn2, Hcn3* and *Hcn4* mRNA expressions in the DRGs of WT and *Hcn3^-/-^* mice. The mRNA levels of *Hcn1 and Hcn2* were unaltered between genotypes, whereas *Hcn3* mRNA expression was not detectable in DRGs of *Hcn3^-/-^* mice. *Hcn4* mRNA was not expressed in DRG neurons of WT and *Hcn3^-/-^* mice. Data are means ± SEM relative to *Hcn1* expression in WT mice from *n* = 5 mice per genotype.

**Supplementary Table 1.** Raw data of double *in situ* hybridization for *Hcn3* and established markers in thoracic DRGs. Data corresponds to the quantitative summary presented in Figures 1 and 2. The number of cells was counted from DRG sections of n = 2–3 mice.

| **Marker** | ***Hcn3* population** | **Marker population** | **# DRG sections** | **Total # neurons** |
| --- | --- | --- | --- | --- |
| *Rbfox3* | 4048 | 6612 | 77 | 6612 |
| *Vglut3* | 99 | 128 | 5 | 589 |
| *Ntrk2* | 37 | 82 | 6 | 395 |
| *Ntrk3* | 98 | 102 | 4 | 382 |
| *Pvalb* | 57 | 61 | 4 | 343 |
| *Kcnt1* | 200 | 284 | 4 | 429 |
| *Calca* | 118 | 129 | 4 | 414 |
| *Trpm8* | 63 | 63 | 7 | 669 |

**Supplementary Table 2.** Raw data of double *in situ* hybridization for *Hcn3* and established markers in lumbar DRGs. Data corresponds to the quantitative summary presented in Figures 1 and 3. The number of cells was counted from DRG sections of n = 2–3 mice.

| **Marker** | ***Hcn3* population** | **Marker population** | **# DRG sections** | **Total # neurons** |
| --- | --- | --- | --- | --- |
| *Rbfox3* | 2880 | 4854 | 65 | 4854 |
| *Vglut3* | 70 | 91 | 5 | 405 |
| *Ntrk2* | 33 | 47 | 4 | 436 |
| *Ntrk3* | 95 | 99 | 5 | 326 |
| *Pvalb* | 49 | 51 | 4 | 226 |
| *Kcnt1* | 95 | 124 | 4 | 218 |
| *Calca* | 22 | 25 | 4 | 114 |
| *Trpm8* | 37 | 75 | 10 | 736 |
